# Development of the WHO Caregiver Skills Training Program for Developmental Disorders or Delays

**DOI:** 10.3389/fpsyt.2019.00769

**Published:** 2019-11-11

**Authors:** Erica Salomone, Laura Pacione, Stephanie Shire, Felicity L. Brown, Brian Reichow, Chiara Servili

**Affiliations:** ^1^Department of Psychology, University of Milan-Bicocca, Milan, Italy; ^2^Department of Mental Health and Substance Abuse, World Health Organization, Geneva, Switzerland; ^3^Department of Psychiatry, Division of Child and Youth Mental Health, University of Toronto, Toronto, ON, Canada; ^4^Special Education and Clinical Sciences, College of Education, University of Oregon, Eugene, OR, United States; ^5^Research and Development Department, War Child Holland, Amsterdam, Netherlands; ^6^Amsterdam Institute of Social Science Research, University of Amsterdam, Amsterdam, Netherlands; ^7^Anita Zucker Center for Excellence in Early Childhood Studies, University of Florida, Gainesville, FL, United States

**Keywords:** neurodevelopmental disorder, developmental delay, disability, caregiver skills training, parent-mediated, nurturing care

## Abstract

Globally, 52.9 million children under the age of 5 experience a developmental disability, such as sensory impairment, intellectual disability, and autism spectrum disorders. Of these 95% live in low-and-middle-income countries. Most of these children lack access to care. In light of the growing evidence that caregivers can learn skills to support their children’s social communication and adaptive behavior and to reduce their challenging behavior, the World Health Organization developed a novel Caregiver Skills Training Program (CST) for families of children with developmental disorders or delay to address such treatment gap. This report outlines the development process, content, and global field-testing strategy of the WHO CST program. The CST program is designed to be feasible, scalable, and adaptable and appropriate for implementation in low-resource settings by nonspecialists. The program was informed by an evidence review utilizing a common elements approach and was developed through extensive stakeholder consultation and an iterative revision process. The program is intended for a global audience and was designed to be adapted to the cultural, socioeconomic, geographic, and resource context in which it is used to ensure that it is comprehensible, acceptable, feasible, and relevant to target users. It is currently undergoing field-testing in more than 30 countries across all world regions.

## Introduction

Around 250 million children, or 43% of all children younger than 5 years, in low- and middle-income countries (LMICs) are at higher risk of not reaching their developmental potential due to stunting, poverty, and disadvantage (1). In 2016, it was estimated that globally 52.9 million children younger than 5 years experienced a developmental disability, such as sensory impairment, intellectual disability, and autism spectrum disorders, and 95% of them lived LMICs (2). Most of these children lack access to care. Convincing evidence shows that parents are able to learn skills to effectively promote their children’s development and positive behavior (3).

The WHO is seeking to address this treatment gap and strengthen access and quality of health services and supports to families (4). In this context, a novel program to strengthen caregiving skills for families of children with developmental delays and disorders was developed. The program stems from the need to provide nurturing care to all children, a principle called for by both the WHO Global Strategy for Women’s, Children’s and Adolescents’ Health and the United Nations Sustainable Development Goals on access to high-quality early childhood development. As such, the program is based on the assumption that caregivers of children with developmental disorders or delays can and should be specifically supported in both tapping into their existing competences and developing new skills that can foster their child’s learning, social communication, and adaptive behavior. This report outlines the development process and content of this novel caregiver-mediated intervention for neurodevelopmental disorders and delays, followed by considerations for necessary cultural and contextual adaptations, and the need for future evaluations of efficacy and cost-effectiveness.

## Development of the Who Caregiver Skills Training Program

Central to the development of caregiver skills training (CST) was the requirement that the target beneficiaries of the intervention were children with heterogeneous developmental difficulties and there was a need to promote scale-up in LMICs with the use of briefly trained nonspecialists. The program was designed to adopt a family-centered approach that fit within a stepped-care model, where CST is to be integrated into existing maternal, child, and family health and social services. In terms of the estimated economic impact of implementing the program, it is notable to mention that the requirements for training the intervention providers, who are nonspecialists, are much lower than in many other comparable fee-based intervention programs requiring specialist providers; thus, the implementation costs are expected to be lower. The impetus for the development of the CST program lies within the WHO’s mhGAP Intervention Guide for mental, neurological, and substance use disorders in nonspecialized health settings, which since 2010 recommends parent skills training for developmental disorders or delays, if available (5). However, at the time this recommendation was made, no such program was freely available to use, adapt, and scale up globally. This, along with increasing global interest reflected in the World Health Assembly’s resolutions on mental health in general and the one on comprehensive and coordinated efforts for the management of autism spectrum disorders specifically (6), created the momentum for WHO to develop the CST for a global audience. Among the key priority areas of action for WHO and partners, the development and facilitation of access to competency-based training materials for a range of care providers including parents were established in a subsequent WHO technical consultation.

The formative process to develop CST consisted of a systematic review and meta-analysis and an extensive expert consultation. The systematic review included the analysis of implementation processes and component analysis using metaregression techniques with the aim of identifying key components and characteristics of efficacious interventions (7, 8). The review findings indicated that caregiver-mediated interventions for families of children with autism spectrum disorder who are early communicators and of children with intellectual disabilities can be effectively delivered by nonspecialists in community settings and that improvements in both child developmental and behavioral outcomes and family well-being could be achieved even with low-intensity programs. It also showed that programs that included behavior management techniques and strategies to improve caregiver coping were more effective than programs that did not contain this content. Additionally, programs that used a combined delivery format of group and individual sessions showed a greater impact than either format used in isolation on reduction of challenging behaviors.

As starting point for the development of CST, the results of the systematic review and meta-analysis were examined and discussed by global leaders with experience in parent-mediated interventions from diverse professional, geographic, and cultural backgrounds at a meeting at WHO Headquarters. The meeting included representatives from 21 countries across all six populated continents with a majority of representatives from LMICs, according to World Bank Classification. Representatives included academic leaders, clinicians, foundation leadership, practitioners, and caregivers (beneficiaries). These representatives were asked to advise on content and structure of the intervention, help address acceptability and feasibility concerns, and identify capacity building strategies.

A few aspects of the program were carefully considered at the intervention design stage: (a) engagement of other caregivers and family members; (b) promotion of caregivers’ well-being and acceptance of the child’s difficulty as prerequisites to learning skills; (c) potential demand-side barriers to accessing the program, including stigma; and (d) heterogeneity of children’s developmental and health profiles, with particular regard to the high prevalence

A modular approach was suggested with “core” individual and group sessions followed by additional optional sessions, to address specific needs and considering availability of resources. The content of the core sessions was recommended to include promoting joint engagement, promoting spoken and nonverbal communication to request and share attention, reducing challenging behavior, teaching skills for daily living, and promoting caregiving well-being. Additional topics that were suggested as important included strategies tailored for children who have minimal spontaneous spoken language and additional materials on caregiver well-being and comorbid conditions. Alternative scripts for stories and role plays are provided to enable further tailoring of the content to the needs and priorities of families.

The program was designed to include a combination of group sessions in community centers, health centers or schools and individual sessions in caregivers’ homes. The group format for most of the intervention keeps implementation costs (human resources, travel time) to a minimum and promotes peer support among caregivers, while the individual home visits allow the intervention to be responsive to each family’s needs by tailoring guidance to the caregiver and child characteristics. The home visits serve in fact the purpose of setting goals for the child’s development based on the assessment of skills in a naturalistic setting and the identification of family priorities; tailored coaching on the intervention strategies is then provided, and goals are periodically reassessed. Home visits are also critical for rapport building with the whole family and identification of additional family needs requiring referral to other services or professionals.

A cascade training and supervision model was decided on as a critical element for effective and cost-effective implementation of CST by nonspecialist providers (such as nurses, community health workers, and peer caregivers) at the health facility or community level, or in schools, allowing for program scale-up. To effectively support the implementation of CST by nonspecialist providers, especially in lower-resource settings, a continuous support and supervision model was incorporated.

Given the global lack of service providers and long wait times, which would create an unnecessary delay in access to needed treatment, the inclusion and exclusion criteria for the target beneficiaries of the intervention were chosen to be as inclusive as possible, and it was decided that a diagnosis should not be required. The target group for the program was therefore identified as that of caregivers of children aged 2 to 9 years presenting with a developmental disorder or delay. A further consideration was that creating a program exclusively for families of children with a formal diagnosis of developmental disorder could be a barrier to families’ engagement due to lack of diagnostic services, delay in diagnosis, and stigma. The wide age range was decided on because children are often identified late, particularly in low-resource settings, and a program for caregivers of younger children would need to be offered in partnership with early intervention programs, which may not be yet in place. Other principles agreed on included the need to carefully consider the optimal intensity of the program in terms of number and duration of sessions. Considerable attention was paid to retention strategies designed to decrease the risk of dropout and increase engagement of families, such as regular phone calls, text messages, and provision of refreshments.

## Contents and Structure of WHO CST

With regard to the selection of intervention goals, the program was designed to target (a) the child’s development, specifically in regard to promoting social communication and adaptive skills and reducing disruptive and challenging behavior; (b) the caregiver–child relationship; (c) the child’s participation and inclusion in daily home and community activities; and (d) the caregiver’s role and functioning, by promoting self-confidence, parenting skills and knowledge, and coping skills and psychological well-being. The program was designed to include nine core modules for group sessions aimed at training caregivers in the use of strategies that target key domains of child and caregiver functioning (**Table 1**), complemented by three home visits; three optional group session modules were also developed. Home visits are scheduled before the first group session, before the midpoint of the program and after the last group session, with the purpose of tailoring the intervention to the families’ individual environments, goals, and needs.

**Table 1:** WHO caregiver skills training: structure.

Module	No. of sessions	Notes
Engagement	2	Dedicated module. Sessions can be delivered separately or as a single session
Play and home routines	1	Dedicated module
Communication	2	Dedicated module
Behavior management	2	Dedicated module
Adaptive behavior	1	Dedicated module.
Caregiver self-care and ongoing practice	1	Dedicated module. Self-care activities are also embedded in each of the other core sessions.
Minimally verbal children	3	Optional module
Comorbid conditions	1	Optional module. Informative text is also embedded in each of the core sessions.
Caregiver well-being	3	Optional module

A major recommendation from the expert consultation was the requirement to appropriately address the heterogeneous needs of children and families, given the broad target group. To allow the intervention to be tailored to children and families’ needs, a threefold strategy was devised: first, goal-setting activities were embedded throughout the program to ensure that the intervention could be tailored to the child’s developmental level and the family’s priorities and flexibly adjusted as the child progresses. To this end, during the first home visit, the nonspecialist provider works jointly with each family to identify two “target routines” (semistructured opportunities for learning and development, as explained below) that match the interventionist’s observations about the child’s needs and developmental level, while meeting the family’s priorities and daily activities. These target routines are regularly revised during the program to ensure that they continue to be appropriate. The second individualized component of the program consists of one-to-one coaching provided to caregivers both during group sessions, through role-play activities, and at the home visits, during live interaction with the child. The coaching component facilitates learning of the intervention strategies taught during the group sessions, ensuring that the caregiver is given clinically sensitive, individualized feedback to develop those skills and competencies that particularly suit the child’s needs. Lastly, optional modules with a focus on (a) children who have minimal spontaneous spoken language, (b) those with other comorbid conditions, and (c) caregiver well-being were made available to ensure that comorbidities and other co-occurring needs could be addressed.

A suite of materials for field testing was developed, including (a) intervention manuals and user-facing documents (session-by-session facilitator guides, facilitator home visits guide and goal setting form and session-by-session participant booklets); (b) materials developed to assist countries in the planning and adaptation phases (planning guidance, planning meeting materials, adaptation and implementation guidance and materials); (c) materials for training master trainers and facilitators (training of trainer [ToT] course and supervision models); and (d) materials to record, monitor, and assess processes and outcomes in the prepilot and pilot-testing phases (monitoring and evaluation framework).

### Theoretical framework and methodology

The program’s theoretical foundation and methodology are informed by principles of applied behavior analysis, developmental science, social communication interventions, positive parenting, and self-care methods. The primary program targets are defined as increased spontaneous nonverbal and verbal communication and increased time in shared engagement, and secondary targets include reduced child challenging behavior, improved caregiver coping skills and psychological well-being, and improved family functioning. The program was developed with an additional aim of facilitating stigma reduction against persons with developmental disorders and promoting increased inclusion and community participation of these children.

The common thread to accomplish the program’s goals is the shaping of common activities into regular shared caregiver and child routines that become opportunities for learning and development. Caregivers are encouraged to practice interacting with their child within both “home routines” (setup within activities done regularly, such as eating, dressing, caring for animals, tidying and going into the community to run errands, pick up siblings, or enjoying being outside) and “play routines” with toys and recycled materials (e.g., cups or cardboard boxes) that are easily available to the family. Over the course of the program, caregivers are taught intervention strategies derived from principles of Naturalistic Developmental Behavioral Interventions and principles of applied behavior analysis for neurodevelopmental disorders. Naturalistic Developmental Behavioral Interventions, which include JASPER (9, 10) and PRT (11), are intervention methods derived from principles of both behavioral learning and developmental science (12). At the core of the CST program is therefore, on the one hand, the developmental principle that children’s development is favored within developmentally appropriate (13, 14), affectively rich (15) learning contexts. Within that framework, such contexts become learning opportunities where the adult follows the child’s choices of materials and activities (16), promptly responds to and expands child’s communication, and actively transforms activities into motivating play or daily living routines (17), with an emphasis on turn taking, affect, and developmental appropriateness of materials and tasks. On the other hand, CST uses incidental teaching techniques incorporating elements of the science of learning including modeling, shaping, chaining, prompting, and differential reinforcement within the context of natural stimulus conditions of everyday environments (18).

Such teaching techniques are used within a naturalistic framework, with the use of natural rewards (19), use of child-preferred options (20), and reinforcement of approximations and communicative attempts. Principles of functional analysis derived from applied behavior analysis are also taught within CST, and strategies to support the child’s regulation are illustrated. Emphasis is given on the use of a variety of environmental strategies to support child’s engagement, promote spontaneous communication, and reduce dysregulated or challenging behavior (9). In this respect, foundational strategies include guidance on the adult’s positioning (in front of and at the child’s level, with the activity in between them) and offering the child to choose among a range of developmentally appropriate materials. Other “environmental strategies” include strategically arranging the setting (by controlling access to materials of interest and using materials that either require assistance, are provided in small quantities at a time or are in sight but out of reach) and creating affectively salient contexts that require the child’s active participation, such as playful obstruction, expectant waiting, or violating an established routine.

An additional desirable characteristic of this approach is that it facilitates caregivers’ involvement because these naturalistic teaching strategies can be easily implemented within the family context, by transforming everyday activities (meals, bath time, outings, etc.) into child-led learning opportunities with a clear structure (routine building) that work toward prespecified developmentally appropriate targets (goal setting). Exposing the child to multiple learning experiences targeting the same skills in different real-life contexts (as opposed to structured trials with artificial stimuli) is shown to predict greater generalization of skills, tolerance of real-world distractions, and reduced dependence on prompting (21).

Furthermore, the approach favors and reinforces the parent’s natural role, since caregivers are encouraged to commit to daily moments of interaction with the child within regular activities that would be carried out regardless, instead of being asked to take up the role of a “therapist” carrying out additional structured tasks, resulting in happier, less stressful interactions and more positive communication styles (22). Caregivers of children with developmental disabilities experience higher stress and distress than parents of typically developing children (23, 24). Furthermore, parent distress predicts child outcomes and outcomes from behavioral parenting interventions (23, 25, 26). Therefore, the approach emphasizes the importance of parent self-care throughout core sessions, including introduction of relaxation exercises. An optional module on caregiver well-being is also incorporated, which draws on strategies from acceptance and commitment therapy, an empirically based approach that has shown promise in improving parent and child outcomes in families of children with disabilities (27–30).

To provide a practical example of how within CST caregivers are taught routine building, we illustrate below how to establish a routine in the context of a caregiver and child dyad taking items out of a shopping bag. First, the adult would need to identify which specific actions constitute, on that occasion, the steps that are appropriate for them and their child to follow (e.g., for a child who can use word combinations and combine different materials and play actions, these may include labeling and sorting groceries into different containers or areas in a cupboard, while for an early learner, taking each item out of the bag while the adult provides simple language may be enough). Then, the adult would make sure to assume an active but balanced role, rather than solely asking the child to complete actions or, conversely, dominating the interaction completely by leading all the steps. A balanced active role would therefore entail taking turns with the child in completing each step; these may mean alternating on doing the same action (direct imitation), or steps may be different for the child and the adult, such as the child taking items out and the adult putting them into a cupboard or container. A strong focus would be given on promoting child engagement by making the routine affectively salient (e.g., showing enthusiasm and pairing actions with display of affection or adding “fun elements,” such a song). The underlying goal, in line with the programs’ primary targets, would be to promote the child’s communication by giving the child the opportunity to communicate (staying silent and looking expectantly at the child on the child’s turn) and responding and expanding the child’s communicative signals (gestures, sounds, words, or eye gaze) by combining a gesture and language at the child’s level (e.g., to describe each item they remove from the bag or to label relevant actions, such as “out!”). Positive attention, imitation, and social rewards (praise) would be provided contingently to the child’s actions or approximations, in order to reinforce the performance.

The evidence-based principles illustrated above are taught to caregivers using accessible language as *key messages* (general psychoeducational messages about developmental disorders and delays) and *tips* (hands-on strategies and skills for interacting with the child). The latter are shown during the group sessions through adult-learning techniques such as group discussions, modeling, and guided role playing (**Table 2**). Illustrated booklets with the key messages and tips are provided to participants at each group session. In addition, the one-to-one provider-to-caregiver coaching provided during the home visits (prior to the first group session, midway, and at the end of the program) is an opportunity to give more emphasis on strategies that are most relevant and suitable for each caregiver–child dyad.

**Table 2:** WHO caregiver skills training: activities and learning methodology.

Setting	Activity	Objectives	Methodology
Group session	Wellness activity	To promote and practice caregiver self-care	Diaphragmatic breathing relaxation exercises have been shown to reduce anxiety (31)
	Review of previous contents and home practice	Reflection on prior learning; self-reflection; problem solving: learners share personal experiences and knowledge with each other	Goal setting, self-reflection, and problem solving are evidence-based instructional strategies for adult learners (32)
	Discussion of a story	Learning through a caregiver’s story followed by group discussion allows caregivers to see how skills and strategies can be implemented in day-to-day life. Caregivers in the stories model acceptance and normalize difficult emotional experiences common to caregivers	Integration of learning through story (33)
	Presentation of new content	Caregivers learn skills and strategies they can practice at home in small illustrated steps	Knowledge scaffolding: skills and strategies are broken down into small steps (34).
	Demonstration	Learners see how skills and strategies can be applied in routine activities with children	Demonstration followed by practice is an effective adult learning strategy (35)
	Role play	Rehearsal of skills and strategies with other learners in an ideal setting before practice at home	Structured role play (simulation) with feedback can improve skills and increase confidence (36)
	Plan for home practice	Goal setting for how knowledge and skills will be applied at home	Goal setting and self-reflection on personal goals are effective adult learning strategies, and home practice is a common element in caregiver-mediated interventions (37, 38)
Home visit	Review of home practice and goal setting	Learners set own goals for their child	Setting goals for the child is a common element in caregiver-mediated interventions (37)
	Coaching	Modeling of skills; reinforcing strengths; providing immediate feedback	*In vivo* coaching is used to refine skills and is a common element in caregiver-mediated interventions (37, 39)

### Fidelity of Implementation Criteria

In line with most evidence-based interventions, the CST intervention package includes measures of fidelity of implementation. As delivering CST involves both proficiency in leading group sessions and mastering the direct use of intervention strategies with children, competencies in these domains are assessed, respectively, with an adaptation of the ENACT (ENhancing Assessment of Common Therapeutic) scale (40) and a bespoke Adult/Child Interaction Fidelity Scale (WHO CST Team, unpublished). The Adapted ENACT (WHO CST Team, unpublished) includes assessment of verbally illustrating and modeling use of strategies, facilitating group discussion, coaching caregivers within role play, and sensitive reflection and feedback provision. Ratings can be done on video recordings or live observations of group sessions and home visits representing at least 25% of the program content. The Adult/Child Interaction Fidelity Scale is rated on 15-minute video recordings of interaction within either home activities or play contexts and covers use of strategies to support regulated behavior and promote engagement and communication and child’s learning of new skills. Since fidelity of implementation is key to optimal child outcomes (41–43), the WHO CST Team encourages the assessment of fidelity in all phases of field testing.

## Cultural and Contextual Adaptation of Who Cst

As a global program, WHO CST was developed to be adapted to the cultural, socioeconomic, geographic, and resource context in which it is used. Adaptation refers to the systematic modification of an intervention to ensure that it is comprehensible, acceptable, feasible, and relevant to target users (44). There is evidence that culturally and contextually adapted programs are effective and improve feasibility (45–48). The implementation package for the CST program outlines the objectives and process of adaptation in detail using the Bernal Framework, a method for coding adaptation of interventions (49). This framework uses the ecological validity model, which consists of eight dimensions: language, persons, metaphors, content, concepts, goals, methods, and context (50). The goals of adaptation are to ensure that, first, the program content is comprehensible, culturally acceptable, and relevant to local participants; second, the program is responsive to the local socioeconomic, political, and cultural context, and third, it is delivered in a way that meets participants’ needs. The adaptation process aims to maximize accessibility, feasibility, and acceptability and reduce foreseeable barriers to participation. As part of the development phase, effort was made to reduce the need for cultural adaptation by limiting the use of cultural symbols and phrases and utilizing more universal symbols and phrasing, using plain language whenever possible, avoiding Western biases such as toward individualism or consumerism, and aiming to ensure that the program is consistent with the reality of participants in low-resource settings. Illustrations in participants’ booklets were designed to represent multiple cultures and socioeconomic contexts and to reflect the intended global audience. Adaptation guidance was created and included as part of the CST toolkit. The suggested process includes creation of a local adaptation team, formal consultation with an adaptation advisory group of community stakeholders, and adaptation framework, guidance, and documentation form. Adaptations to the program can be made to the program content (aspects of the nonspecialist provider guides and participants booklets) and to the program process (e.g., frequency and setting of group sessions, provision of additional services, supervision and training, etc.). Recommended adaptations to program materials include (i) translation into the local language, ensuring language use (vocabulary, phrasing, verbal style, etc.) is culturally appropriate, literacy level is consistent with that of the intended participants, and technical terms are explained in culturally and linguistically appropriate terms; (ii) changing aspects of content, including the names of characters in stories and role plays so that they are familiar to participants, adding local stories or examples; (iii) adaptations to improve feasibility and acceptability, including choosing an appropriate group session schedule (weekly/biweekly, daytime, or after hours), providing child care, refreshments, or culturally appropriate additional activities.

## Global Field-Testing Initiative

An iterative process of revisions that incorporated inputs from the first stakeholder workshop and a second external expert review resulted in the finalization of program materials (WHO CST Test-Run Version). This version of the program was prepiloted for the first time with a group of caregivers of preschoolers with autism spectrum disorder and co-occurring intellectual disability in Northern Italy. The objective of the test run was the preliminary assessment of feasibility and acceptability of key delivery components and methods of the program prior to making available the materials for global field testing. Group sessions and home visits were led by a WHO CST Team member who contributed to the development of the program and translated the materials in Italian (ES), assisted by a local clinical psychologist with expertise in disability and parenting programs. The choice of specialist, rather than nonspecialist, providers was deemed necessary to allow for live troubleshooting, even though the program had been ultimately designed to be delivered by nonspecialists. The post-program qualitative–quantitative evaluations with caregivers and nonspecialist providers informed a light-touch revision of materials aimed at improving the provider’s manuals by (1) streamlining redundant content, (2) editing the instructions for activities (e.g., demonstrations) that had been reported as lacking clarity, and (3) formatting the text to improve readability. The revised program materials were then made available for field testing in 2016 (WHO CST Field Test version 1.0). Data derived from the evaluation of feasibility and acceptability of the test run implementation and those collected from consultation meetings, master training courses, and prepilot testing of the WHO CST Original Version in the first countries involved in the global CST field-testing initiative, such as Ethiopia (51), were collated. The adaptation database that was created informed the development of a revision of the program materials for global field testing (WHO CST Field Test Version 2.0). This revised version comprises reduced and simplified contents organized in sessions of 2 to 2½ hours. The complete suite of materials for field testing includes intervention manuals, training and supervision models, monitoring and evaluation framework.

To date, there are 30 active field-testing sites, representing all WHO world regions, African (n = 4), Americas (n = 9), Eastern Mediterranean (n = 6), European (n = 4), Southeast Asia (n = 2), and Western Pacific (n = 5). Official field-test versions of the package are now also available in Spanish, and translations are in process in multiple other languages. Participating sites progress through four phases outlined in a monitoring and evaluation framework for field testing, consisting of (1) planning and adaption, (2) ToT and post-ToT practice, (3) prepilot field testing, and (4) pilot testing. Input from field testing is being collected in order to contribute to the development of the final version of the CST package, which will be made available on the WHO website.

A survey of adaptation processes and contents among sites participating in the field testing is underway. Preliminary data from 28 sites indicate that the majority of sites (n = 26) reported having adapted the program to the local context, mostly with minor measures: of these, 76% were adaptations of content (e.g., language use, idioms), 17% were adaptations to improve feasibility (e.g., child care), and 7% were adaptations of processes (e.g., frequency of group sessions). Minor changes to content included changes to names of characters, idioms, language use, aspects of stories, objects, style of character dialogues in the stories, the addition of psychoeducational messages particularly relevant to the context (e.g., addressing local myths about developmental disorders and delays), and modification of illustrations for ethnic, cultural, and contextual reasons. Changes to program process to support attendance included providing child care for group sessions, weekly peer support phone calls, refreshments, small gifts, post-program celebration, and additional outdoor self-care activities for caregivers. An adaptation for a low-resource, low-literacy setting was conducted in Ethiopia in consultation with community stakeholders. Adaptations included modification of activities that required writing, simplification of provider demonstrations and participant booklets, additional information on addressing expectations of a cure and discouraging physical punishment, removal of the picture schedule component, and increased emphasis on use of gestures. In pilot testing, the locally adapted WHO CST program was found to be acceptable and feasible for caregivers (51). The program was also adapted for delivery by family volunteers in rural Pakistan using a tablet-based application that serves as a training, intervention delivery, and monitoring tool. The key program contents (key messages and strategies) were incorporated into “real-life” narratives of the lives of three local children with developmental disorders, their family members, and other supporting characters with graphic images representing each character, which are used to voice narrative scripts (52). Adaptation and piloting of the program in high-income settings are also underway, including in Italy (53), Canada, and the United States.

The field-testing phase and, in particular, data derived from the planning and engagement workshops with stakeholders will provide additional insight into opportunities for integrating the support to caregivers into existing community-based services or programs. Additional studies, evaluating cost-effectiveness, component analysis, alternative delivery methods, and dosage, will also be beneficial. Findings from the field-testing and future research studies can be used to inform future implementation and research on scalable, sustainable interventions for children with developmental disorders or delays.

## Data Availability Statement

The datasets generated for this study are available on request to the corresponding author.

## Ethics Statement

Ethical review and approval was not required for the development of the caregiver skills training program described in this article, in accordance with the local legislation and institutional requirements. However, local ethics approval was obtained at field-testing sites in accordance with the local legislation and institutional requirements. The participants provided their written informed consent to participate in this study.

## Author Contributions

CS conceived of and led the CST initiative. BR led the meta-analysis and systematic reviews, with support of CS, which helped establish the empirical evidence and theoretical framework of the CST program. LP, ES, SS, and FB contributed to the drafting of the CST program materials and field-testing materials; ES and LP supported field-testing sites with supervision by CS. AS provided institutional support. ES and LP led the writing of the paper with input from all authors.

## Conflict of Interest

The authors declare that the research was conducted in the absence of any commercial or financial relationships that could be construed as a potential conflict of interest.
